# Development of the Sm14/GLA-SE Schistosomiasis Vaccine Candidate: An Open, Non-Placebo-Controlled, Standardized-Dose Immunization Phase Ib Clinical Trial Targeting Healthy Young Women

**DOI:** 10.3390/vaccines10101724

**Published:** 2022-10-15

**Authors:** Marília Santini-Oliveira, Patrícia Machado Pinto, Tatiane dos Santos, Mônica Magno Vilar, Beatriz Grinsztejn, Valdilea Veloso, Elan C. Paes-de-Almeida, Maria A. Z. Amaral, Celso R. Ramos, Miryam Marroquin-Quelopana, Rhea Coler, Steven Reed, Marcia A. Ciol, Wilson Savino, Juçara de Carvalho Parra, Marília Sirianni dos Santos Almeida, Miriam Tendler

**Affiliations:** 1 Evandro Chagas National Institute of Infectology, Fiocruz, Rio de Janeiro 21040-360, Brazil; 2 Laboratory of Experimental Schistosomiasis, Oswaldo Cruz Institute, Fiocruz, Rio de Janeiro 21045-900, Brazil; 3 Department of Basic Sciences, Fluminense Federal University, Nova Friburgo 28625-650, Brazil; 4 Program for Development of the Mata Atlântica Campus, Fiocruz, Rio de Janeiro 21045-900, Brazil; 5 Fenix Biotec Treinamento SS LTDA, São Paulo 05591-090, Brazil; 6 Department of Global Health, University of Washington, Seattle, WA 98195, USA; 7 HDT Bio, Seattle, WA 98102, USA; 8 Department of Rehabilitation Medicine, School of Medicine, University of Washington, Seattle, WA 98105, USA; 9 Laboratory on Thymus Research, Oswaldo Cruz Institute, Fiocruz, Rio de Janeiro 21040-360, Brazil; 10 Brazilian National Institute of Science and Technology on Neuroimmunomodulation, Oswaldo Cruz Institute, Fiocruz, Rio de Janeiro 21040-360, Brazil; 11 René Rachou Institute—Fiocruz/Minas, Fiocruz, Belo Horizonte 30190-002, Brazil

**Keywords:** vaccine, schistosomiasis, phase Ib clinical trial, toxicology, pregnant rabbits, Sm14 protein

## Abstract

We report the successful closure of Phase I clinical trials, comprising Phases Ia and Ib, of the vaccine candidate against human schistosomiasis: the *Schistosoma mansoni* 14 kDa fatty acid-binding protein (Sm14) + glucopyranosyl lipid A in squalene emulsion (GLA-SE). Shown here are the results of Phase Ib, an open, non-placebo-controlled, standardized-dose immunization trial involving 10 healthy 18–49-year-old women. Fifty micrograms of the Sm14 protein plus 10 µg GLA-SE per dose was given intramuscularly thrice at 30-day intervals. Participants were assessed clinically, biochemically, and immunologically for up to 120 days. In preambular experiments involving vaccinated pregnant female rabbits, we did not find any toxicological features in either the offspring or mothers, and the vaccine induced adaptive immunity in the animals. In women, no adverse events were observed, and vaccination induced high titers of anti-Sm14 serum IgG antibody production. Vaccination also elicited robust cytokine responses, with increased TNFα, IFNγ, and IL-2 profiles in all vaccinees on days 90 and 120. The completion of Phase I clinical trials, which were performed to the highest standards set by Good Clinical Research Practice (GCP) standards, and preclinical data in pregnant rabbits enabled the vaccine candidate to proceed to Phase II clinical trials in endemic areas.

## 1. Introduction

Schistosomiasis is a parasitic disease that affects populations living in areas with very poor sanitary conditions, where they are infected through contact with contaminated water during their daily work, domestic, or leisure activities. Children are the main target of the infection, which strongly compromises their physical and cognitive development [1,2]. This feature makes schistosomiasis not only a disease resulting from poverty but also a barrier to development in endemic countries. Schistosomiasis is present in tropical areas of America, Medium East, Asia, and Africa, where the impact on public health is catastrophic as a consequence of the size of the territory affected as well as the number of people who become infected. The World Health Organization (WHO) estimates that over 240 million people living in 78 countries are infected, and around 800 million live in areas with a risk of transmission and infection and are part of global programs directed at the effective control of the transmission of schistosomiasis [3]. According to the Ministry of Health, in Brazil, around 1.5 million people live in areas where they are at risk of acquiring the disease [4], yet we still lack a vaccine to protect these populations from the disease.

Presently, the so-called Mass Drug Administration (MDA) program against schistosomiasis is the strategy adopted by WHO, in which populations in endemic areas are treated annually with Praziquantel without a previous diagnosis. This strategy actually led to an improvement with regard to the pathology associated with schistosomiasis [5]. However, the world prevalence is still as high as it has always been, and the Disability-Adjusted Life Year (DALY) value, which is an important tool to assess the impact of diseases, has increased [6].

The Sm14 vaccine candidate was developed at the Oswaldo Cruz Foundation (Fundação Oswaldo Cruz, Fiocruz, Brazil) as a humanitarian vaccine to be used in populations living in areas at risk of infection [7,8,9].

The Sm14 recombinant protein is currently the basis for an antiparasitic molecular vaccine associated with GLA-SE adjuvant and was developed as an anthelmintic bivalent vaccine directed against fasciolosis and schistosomiasis. Sm14 is a member of the fatty acid-binding protein family, originally isolated from *S. mansoni,* as described elsewhere [10,11,12], and has been identified in most parasitic helminths of human and veterinary importance [5,6,7,8,9,10,11,12,13,14,15,16,17].

During the experimental phase of Sm14 vaccine development, focused on the vaccination of outbred animals, it was possible to demonstrate consistent protection against challenge infection with the cercariae of *S. mansoni* and a significant reduction in the adult worm burdens of vaccinated/infected animals, evidenced by the mean values of adult worm loads and additional measures of the distribution of the frequencies of sequential worm burden rates in Swiss-Webster mice [18,19,20,21]. Therefore, one can expect that vaccination with Sm14+GLA-SE in humans may lead to a reduction in worm burdens, reducing reinfection rates and diminishing the aggressive inflammatory response that has been observed following interrupted chemotherapy in children living in high-transmission areas [22,23].

The major objective of the Sm14 vaccine project is to promote health access through a safe and immunogenic vaccine capable of blocking the transmission of the disease. This will prevent populations from needing to receive repeated doses of chemotherapeutic drugs during childhood and youth. The design of the vaccine foresees its large-scale use, combined with the other known control measures, such as chemotherapy with Praziquantel, sanitation, health education, etc., capable of promoting the control of the disease and mitigating the suffering historically produced by this disease in tropical countries.

The Brazilian National Sanitary Surveillance Agency (Anvisa) approved the start of the Clinical Protocol for Phase Ia Study (CE# 990768/10-8) in a non-endemic area of Rio de Janeiro, Brazil, with 20 healthy male volunteers, which was completed with success and validated. The validation was performed with the use of a Data Bank containing all records of the study for calculations and detailed and descriptive statistical analyses of the results [11]. In order to comply with the requirements for a similar study in women, Anvisa requested experiments to be conducted in pregnant experimental animals. In a second round, after the completion of the required testing of the toxicity of the product under investigation, Anvisa approved the Phase Ib clinical trial in the same Brazilian region as the Phase Ia clinical trial, this time with female volunteers. As required by the regulatory agency, the Sm14+GLA-SE formulation underwent preambular testing and was successfully approved in pregnant rabbits.

The step related to the process of vaccination in Phase Ib (with young adult female volunteers) was conducted by the Evandro Chagas National Institute of Infectology (INI/Fiocruz) using the same batches of Sm14+GLA-SE used in Phase Ia.

The immunogenicity induced by the Sm14+GLA-SE vaccine in women was also approached by means of a platform made available by the Access to Advanced Health Institute (AAHI) (previously Infectious Disease Research Institute IDRI), Seattle, USA, where sera and cells collected at different points of the study from volunteers were shipped. The steps needed to test the safety and immunogenicity of the vaccine and the validation of the study were completed and are described in the present work.

## 2. Materials and Methods

### 2.1. Vaccine Antigen and Adjuvant

For the studies carried out in rabbits as well as the clinical trial, the Sm14 vaccine used in the study was produced at the Ludwig Institute for Cancer Research (LICR) and Cornell University, NY, USA, under good manufacturing practice conditions (cGMP). Hermetically closed vials with individual doses (0.55 mL), labeled with the product name, Sm14, lot number (PBR-0057-002), date of manufacture, and “only for investigational use”, were shipped to Brazil under import authorization from the National Council for Scientific and Technological Development (CNPq) and Anvisa. Vials were kept at 2–8 °C under continuous temperature control from the origin at Fiocruz (Rio de Janeiro) until use, with regular inspections to ensure that contents remained liquid and transparent without suspended particles.

The Infectious Diseases Research Institute/Access to Advanced Health Institute (IDRI/AAHI) provided the glucopyranosyl lipid-A adjuvant in squalene emulsion (GLA-SE) in vials containing 0.4 mL at a concentration of 40 µg/mL, also produced under GMP conditions and labeled with the lot number (Part # 0037-09M001) and manufacturing date. Shipping and storage followed the same procedures as described above.

The batches of Sm14 and adjuvant were filled/finished as single doses and mixed at the time of immunization.

To formulate the vaccine antigen dose at the time of administration, 0.4 mL of a 200 µg/mL Sm14 suspension was aspirated using a 1 mL sterile syringe. Subsequently, the volume of the syringe was injected into the vial containing the GLA-SE adjuvant, obtaining a mixture with a total volume of 0.8 mL. After mixing by inverting the vial, 0.5 mL doses containing 50 µg of Sm14 protein and 10 µg of adjuvant were administered by the intramuscular route to both animals and women volunteers. The dose used in these studies was based on a clinical protocol approved by Anvisa for the Phase Ia study in male volunteers [11]. The two components of the vaccine candidate, kept in separate vials, were monitored by Pharmaceutical Product Development (PPD) [24]. Samples were sent to PPD for control every three months in accordance with applicable standard operating procedures and US Food and Drug Administration (FDA) regulations. The stability of the Sm14 GMP lot was tracked by PPD, and an updated “Certificate of Analysis” was issued.

### 2.2. Preclinical Experiments on Safety and Immunogenicity in Pregnant Rabbits and Respective Offspring following Sm14 Vaccination of the Mothers

The study in pregnant rabbits was conducted in accordance with the protocol provided by Anvisa. It was reviewed and approved by the Ethics Committee on Animal Research of Fiocruz (CEUA/Fiocruz) (n° L0063/08—LW 19/2013).

Reproductive toxicity tests were performed with the same vaccine lot further used in Phase I clinical trials, which was produced under current good manufacturing practice (cGMP) conditions by LICR/Cornell University, NY, USA. Vials containing individual doses (0.55 mL, 200 µg/mL) were labeled with the product name (Sm14) and lot number (PBR-0057-002). Vials were kept at +2–8 °C under continuous temperature control until use, with regular inspections to ensure that the contents remained liquid and transparent without suspended particles.

Twenty-four NZW pregnant rabbits, with the same age and weight at the beginning of the experiment, were divided into two groups just after mating: one group comprised twelve rabbits immunized with three equal doses of 50 µg of Sm14 + 10 µg GLA-SE/dose, with a 7-day interval between doses, and the other was the control group, comprising twelve rabbits intramuscularly injected with PBS on the same days. Injections of 0.5 mL/dose for each dose were at the external middle third of the right and left thighs alternately and administered on days 0 (day 9 of pregnancy), 7 (day 16 of pregnancy), and 14 (day 23 of pregnancy). The pregnancy period of these animals is approximately 30 days. Necropsy and anatomopathological evaluations were performed on day 29 of pregnancy.

All animals had the inoculation area shaved and marked before each immunizing dose to further assess possible local reactions, which can include erythema and edema, both being scored according to standard protocols [25]. The daily clinical evaluation of each animal was performed and duly recorded in individual data charts by a veterinarian. Parameters such as the occurrence of abortion, stress level, appetite, feces, and skin/local reactions at inoculation sites were evaluated and measured. Body weight was determined at the beginning and at the end of the study. Food and water consumption was recorded individually every day.

Biochemical and blood cell evaluations were performed following current laboratory practices and included liver, renal, and pancreatic functions, as well as red and white blood cell counts and coagulation parameters.

All animals were anesthetized with thiopental sodium and euthanized by exsanguination. Detailed anatomopathological analyses of all organs were carried out following euthanasia. All animals were subjected to a macroscopic inspection of the organs and cavities. Samples of organs were collected in 10% buffered formalin for later microscopic analysis. The ovaries, uteri, and fetuses were collected and fixed for further analysis, such as number, size, weight, corpora lutea, placenta, and living fetuses. Paraffin-embedded tissue sections were stained with hematoxylin and eosin (H&E) and examined under a light microscope [26].

Serum samples were collected from all rabbits before the start of pregnancy and on the date of euthanasia. The total anti-Sm14 IgG antibody titers in rabbit serum samples were determined by ELISA [27] in accordance with previously described procedures [28]. Briefly, the wells were coated overnight at 4 °C with 100 µL/well of the Sm14 protein solution at 4 µg/mL in carbonate buffer (0.1 M, pH 9.6) and blocked for 1 h at 37 °C, and then serum samples were added in two-fold serial dilutions from 1/100 up to 1/1600. After washing, peroxidase-conjugated goat anti-rabbit total IgG specific to the gamma chain (Santa Cruz Biotechnology, Dallas, Texas, USA) was added to each well, and the plates were incubated for 1 h at 37 °C. Wells were washed, and the reaction was developed by the addition of TMB substrate (T0440, Sigma-Aldrich, St. Louis, Missouri, USA) for 15 min at room temperature in the dark, followed by the addition of Stop reagent (S5814, Sigma-Aldrich, St. Louis, Missouri, USA); subsequently, the OD at 450 nm was measured using a BIORAD device (Model 680).

All statistical analyses were carried out by using GraphPad Prism software version 4.0. All analyses were conducted by applying the two-tailed test, with *p*-values below 0.05 considered to be significant.

### 2.3. Phase Ib Clinical Trial in Healthy Women

The methodology used to conduct the Sm14+GLA-SE candidate vaccine Phase Ib clinical trial in women was essentially the same as described by Santini-Oliveira et al. [11] for Phase Ia with healthy male adults (CE # 990768/10-8-CAAE 0018.0.009.000-10). The protocol for the Phase Ib trial was approved by Anvisa (CE# 0577594139) and by the Evandro Chagas Review Council of the National Institute of Infectious Diseases (INI), Fiocruz (CAAE 09627212.4.0000-5262). In addition, tests were monitored by TECHTRIALS, an independent research company, and data were deposited into a Data Bank [29] with the code NCT01154049.

Twenty healthy female volunteers from a non-endemic area of Brazil (City of Rio de Janeiro) were enrolled and registered with numbers from #1 to #20. Among them, 10 were selected and proceeded to the end of the study. The inclusion criteria comprised a negative pregnancy test, availability during the whole duration of the trial, no evidence of any acute infection, no previous or ongoing serious chronic disease, and normal findings for organ functions, as demonstrated by laboratory tests and the physical examination of the cardio-respiratory and brain/neurological systems, abdominal organs, skin, and lymphatic system. Exclusion criteria included pregnancy/breastfeeding, abnormal hematology or blood chemistry counts or data, any kind of ongoing infection, including infection with the human immunodeficiency virus (HIV), any chronic disease, alcohol/drug abuse, or a history of medication or vaccination in the preceding month. After clinical screening, 10 participants aged 26–48 years (mean = 36.3, sd = 6.8) were selected for the trial, and their demographic characteristics are depicted in Table S1.

Full physical exams and laboratory tests were performed at baseline and on days 0, 7, 30, 37, 60, 67, 90, and 120 (Figure S1). Participants were monitored at the site for up to one hour after vaccination and followed up by phone 20–28 h afterwards. According to Santini-Oliveira [11], all adverse episodes were evaluated in compliance with the International Centers for Tropical Disease Research network (ICTDR) and according to the degree of severity as follows: Level 1 (tolerable with mild discomfort and not interfering with regular activities); Level 2 (discomfort with the impairment of regular activities but not requiring any specific medical assistance or treatment); Level 3 (requiring the discharge of regular activities and medical intervention), and Level 4 (life-threatening). The laboratory at the Evandro Chagas National Institute of Infectology (INI) is certified by the College of American Pathologists (CAP, EUA), and laboratory tests were evaluated based on the INI Standard Values Chart. The study followed FDA regulations [30].

Samples of peripheral blood mononuclear cells (PBMCs) and of sera were collected from all subjects at baseline and on days 0, 30, 60, 90, and 120. Immediately after collection, PBMCs were separated by centrifugation at room temperature in Ficoll-Paque Plus (GE Healthcare, Chicago, IL, USA) and mixed with Fetal Bovine Serum (FBS) (Gibco Life Technologies, Waltham, MA, USA) and 10% DMSO. Cells were subsequently transferred to 2 mL cryovials and frozen at −80 °C in a NALGENE ^TM^ Cryo 1 °C freezing container (Nalgene, Waltham, MA, USA) overnight. On the following day, vials were transferred to liquid nitrogen and later dry-shipped to the Infectious Disease Research Institute (IDRI, Seattle, WA, USA), where they were stored in liquid nitrogen until use.

Sera were also stored in Brazil at −80 °C and transferred on dry ice to IDRI for analysis. At IDRI, sera were stored at −80 °C until experiments were performed. The immunogenicity analyses included checking for isotypes of anti-Sm14 antibodies and cell-mediated responses specifically induced after vaccination with Sm14+GLA-SE. It also included the detection and quantification of intracellular cytokines after the stimulation of PBMCs with purified rSm14. Immune cell responses were measured using the PBMC Luminex Kit for T cell cytokines (Milliplex ^®^MAP, Merck, Burlington, MA, USA) [11]. Isotyping and the correlation between the development and the size of humoral response, measured in terms of IgG against the Sm14 protein, were performed by enzyme-linked immunosorbent assay (IDRI, Seattle, WA, USA) [11,27].

Descriptive analysis using the IBM SPSS v20 statistical package was performed for all variables. Boxplots for all follow-ups assessed whether observations were outside the limits specified for specific laboratory tests. The proportions of adverse events and 95% confidence intervals were calculated using the Clopper–Pearson method [31]. For toxicity grading scales, we used limit values based on published information when available, which included clinical experience and a review of vaccine clinical trials on healthy subjects. Toxicity grading scales were based on the local laboratory reference values when they were defined. Statistical analyses of immunogenicity data were performed using analysis of variance, followed by Tukey’s test for multiple comparisons.

The statistical analysis was performed based on the evaluation of local and systemic reactogenicity observed in the first 36 h after each dose of the vaccine. The results of chemical and hematological exams were compared to reference values in accordance with the Richet Laboratory (LR) criteria and ICTDR, including levels of toxicity as defined by the FDA [30].

## 3. Results

### 3.1. Sm14 Vaccine Candidate Does Not Induce Any Toxicological Effects in Pregnant Rabbits

**Figure 1:**
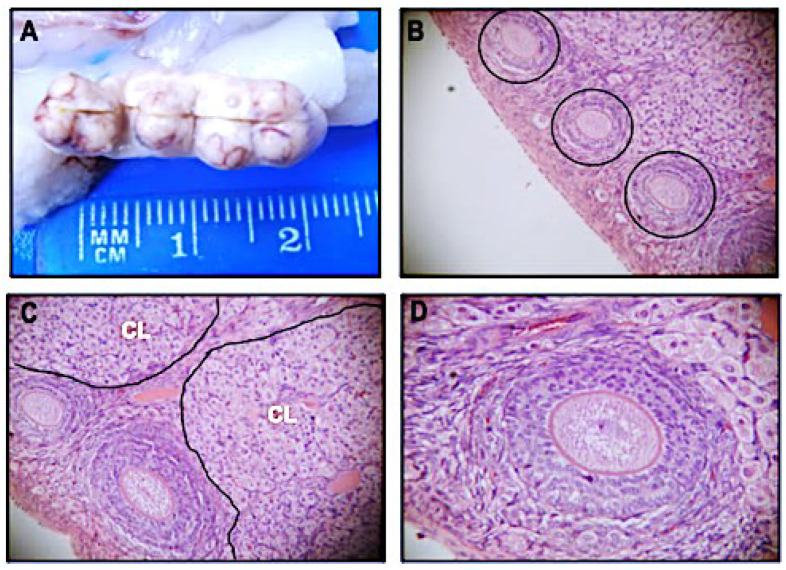
Normal macroscopic and microscopic views of an ovary from an Sm14/GLA-SE-vaccinated pregnant rabbit. Panel (**A**) depicts the normal macroscopic pattern of the ovary, showing a multinodular outer surface and brownish structure with marked vessels. Panel (**B**) shows a microscopic view of follicles in various stages of development in the cortical region (within the black circles). Corpora lutea (CL) can be seen in panel (**C**), whereas a higher magnification of a follicle can be seen in panel (**D**). Sections were stained with hematoxylin–eosin. Scale magnification with 40× (in panel (**B**)), 100× (in panel (**C**)), and 200× (in panel (**D**)) objectives.

The body weights and structures of the analyzed organs of all pregnant rabbits, irrespective of being vaccinated or not, were within normal limits, according to established criteria [32]. Macroscopic analysis of organs and cavities revealed results consistent with the usual aspects of normal pregnant rabbits [33]. The ovaries of all 24 animals were consistent with a normal appearance. All analyzed uteri were comparable to normal pregnant uteri of outbred rabbits according to established criteria [34]. The microscopic results showed that the ovaries of all 24 pregnant rabbits had follicles at different stages of development in the cortical region, typical interstitial stromal glands, and varying numbers of corpora lutea (Figure 1). There was no statistically significant difference in any of the parameters assessed in this study between vaccinated and control animals. Table 1 shows that the duration of pregnancy, the number of implantation sites in the uterus, and the number and viability of pups confirm the absence of maternal and embryo–fetal toxicity in pregnant rabbits vaccinated with Sm14+GLA-SE. The results were also consistent with the morphological and physiological aspects reported for farmed NZW rabbits [32,33,34].

Data are presented as the mean ± standard deviation. The parameters were evaluated by Student’s *t*-test to compare the mean values of groups. In all cases, differences were considered statistically significant at *p*-values of <0.05. No differences were found.

The skin and subcutaneous tissue were removed from the injection sites of the immunized animals. The results of the individual tests showed no significant histopathological alterations in the skin at the immunization site. In the anatomopathological evaluation of the organs, it was observed that all groups, including the control group, had small and scarce foci of calcification in 5% of the cortical and medullary tubules of the kidneys.

**Figure 2:**
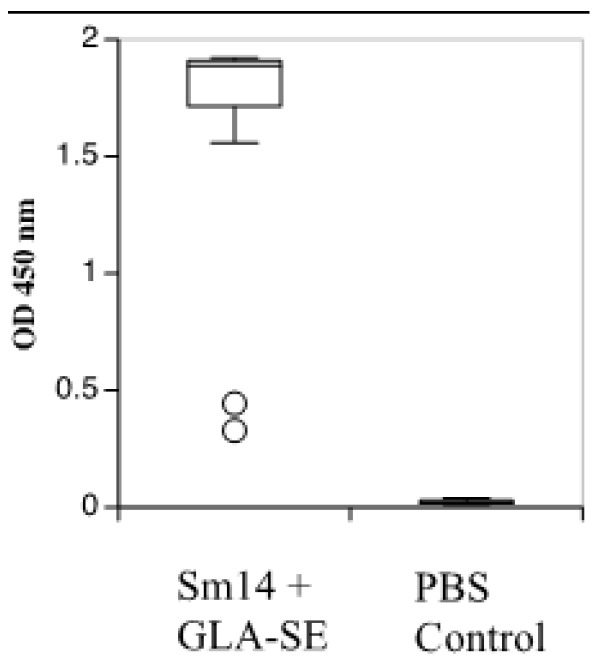
Total anti-Sm14 IgG antibody titers, determined by ELISA, of all rabbit serum samples from the two groups in the reproductive toxicity study. OD: optical density.

Lastly, ELISA was used to analyze sera collected from the animals after the final injection and showed considerably high production of the anti-Sm14 antibody in the sera of vaccinated versus non-vaccinated PBS-treated pregnant rabbits. Anti-Sm14 antibody levels (evaluated by optical density) were clearly high in the vaccinated group as compared to controls (Figure 2).

### 3.2. Sm14 Vaccine Candidate Does Not Induce Any Major Adverse Effects (AEs) in Healthy Women

The certification of Sm14+GLA-SE vaccine safety in the above preclinical study paved the way for a Phase Ib clinical trial of the vaccine in young adult non-pregnant women. The candidate vaccine Sm14+GLA-SE was shown to be safe, with no serious adverse events observed in the participants after any of the three doses. No local reactions at the site of the injection occurred, except for local pain, which affected 9 out of 10 women after the first dose, 5 out of 9 after the second, and 4 out of 10 after the third (all lasting less than 24 h after injection). Only one subject reported involuntary muscle contractions lasting 5 min near the site of injection after the first dose. Table 2 shows the adverse events associated with the vaccination of healthy female volunteers in Phase Ib.

With regard to systemic adverse events, two participants had a fever at D7, and two others had light local hyperemia and a headache at D37, probably related to the vaccine. Physical exams on all days (baseline, 0, 7, 30, 37, 60, 67, 90, and 120) showed no clinical alterations or abnormalities. Laboratory exams did not show alterations that could be classified as toxicity according to the ICTDR criteria, except for ALT and GGT, with few participants showing elevated values within toxicity levels. However, these participants also had elevated values prior to the vaccination (at baseline), and since they had no other abnormal laboratory findings or clinical symptoms, they were considered to be most likely not related to the vaccine. All other single instances of values outside of the normal range were evaluated, and given that no abnormal laboratory findings or clinical symptoms were observed, they were all considered non-clinically significant (that is, most likely not related to the vaccine). These data show that the vaccine produced very few adverse effects, and all observed events were local and mild (Table 3 and Table 4).

### 3.3. Sm14 Does Induce Adaptive Immune Responses in Healthy Women

**Figure 3:**
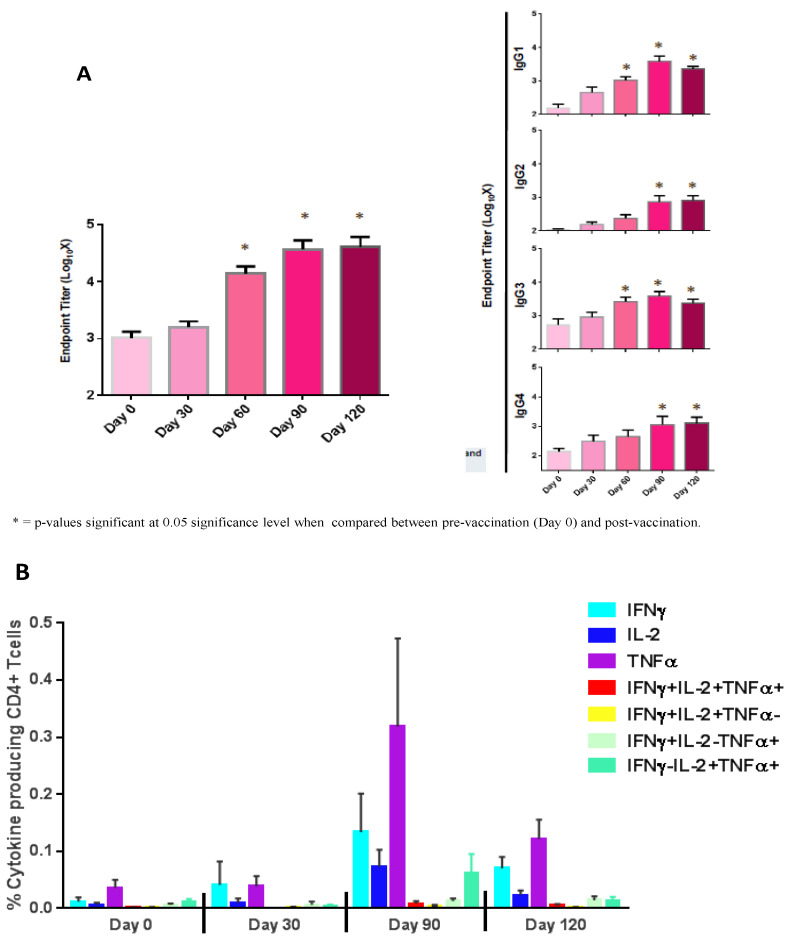
Levels of anti-Sm14 antibody classes and cytokine production stimulated by vaccination. (**A**) ELISA antibody results. (**B**) Percentage of CD4^+^ T cells expressing combinations of IFNγ, TNF_α_, and IL-2 production in CD4+ T cells on days 0, 30, 90, and 120. The comparison of *p*-values to baseline was performed for the vaccinated group (day 0); * *p*-values at the 0.05 significance level.

As occurred with male subjects in Phase Ia [11], the immune response of females vaccinated with Sm14+GLA-SE was characterized by high levels of anti-Sm14 IgG, mainly of the IgG_1_ isotype. Responses were augmented after the second dose. Ninety percent of vaccinated subjects developed higher total specific IgG antibody titers 90 days after vaccination, and these titers remained just as high up to day 120. Compared to the baseline, total specific IgG, as well as IgG_1_ and IgG_3_, increases were statistically significant from day 60 (Figure 3). The levels of specific IgG_2_ and IgG_4_ increased after the second dose and achieved statistical significance from day 90 up to day 120. Additionally, neither general nor specific IgE increases were detected at any time.

Sm14+GLA-SE vaccination elicited robust cytokine responses. Cytokine profiles show increased TNFα, IFNɣ, and IL-2 in vaccinated individuals on days 90 and 120. The analysis results indicated overall comparable responses between male and female cohorts [11] (Figure 3).

## 4. Discussion

Safety is the most important attribute of a vaccine designed for large-scale use in endemic populations. The studies described herein were the basis for legal authorization to start clinical trials for the Sm14 vaccine in Brazil. The preparation was evaluated according to WHO recommendations and guidelines for multiple-dose and reproductive studies that provide preclinical data ensuring the safety of the vaccine formulation [35].

To obtain authorization from the Brazilian health authorities to initiate Phase I clinical trials of the Sm14+GLA-SE anti-schistosomiasis vaccine, Anvisa approved the study to be performed in healthy men living in non-endemic areas for schistosomiasis but did impose a restriction on the engagement of women of childbearing age. A preambular reproductive toxicity test was required in pregnant rabbits to ensure vaccine safety for this population group. The variations observed in laboratory blood tests did not indicate a significant effect on the health of the animals. Coherently, the animals did not show relevant clinical alterations associated with the study, and no macroscopic or histopathological alterations were observed that represented clinical significance. Most of the analyzed parameters were within the normal range of variation, indicating that the vaccine was safe and well tolerated at either dose level, in addition to providing a clear humoral immune response.

These data provided the green light for the Sm14 vaccine clinical test in women of childbearing age to evaluate the clinical steps of the safety and immunological evaluation of the Sm14+GLA-SE anti-schistosomiasis candidate vaccine in healthy volunteer women (clinical trial Phase Ib).

The results obtained in women confirmed the pattern of responses observed in Phase Ia with male adults [11]. Our immunological analysis revealed that Sm14 induced a broad spectrum of immune responses, stimulating both Th1 and Th2 cytokines. However, there was neither a specific nor a general IgE increase, an immunoglobulin associated with the pathogenesis of immediate hypersensitivity reactions because of its ability to bind specifically to high-affinity receptors on mast cells or basophils by the alpha chain of the FC receptor.

## 5. Conclusions

Preclinical assays on pregnant rabbits revealed that the candidate anti-schistosomiasis rSm14-GLA-SE vaccine resulted in no changes concerning the reproductive status or other pregnancy parameters. Moreover, the Phase Ib clinical trial conducted among healthy young adult women confirmed that no serious adverse events were observed after three doses of 50 µg of Sm14 + 10 µg GLA-SE, administered three times at 30-day intervals, in 10 women from a schistosomiasis non-endemic area.

Based on data generated from the clinical studies completed so far, severe toxicity (grade 3 or 4), either local or systemic, is not expected to occur. The administration of the investigational product is anticipated to be well tolerated. So, local reactions (such as pain at the site of injection, erythema, induration, edema, or pruritus) may occur but are usually mild and self-limiting and subside without treatment.

As observed in this trial, adaptive cell-mediated and humoral immune responses (IgG and isotypes, but not IgE), along with many previous preclinical results, strongly support Sm14 as an effective vaccine against *S. mansoni* infection. It may possibly be useful against infections caused by other species of *Schistosoma*.

Clinical studies are a prerequisite for licensing drugs and immunobiologicals by regulatory agencies in most countries. In this way, they are a constituent part of the process of research and development of products for human health. The Sm14+GLA-SE vaccine is at an advanced stage of development, after successfully passing through preclinical stages and clinical Phases Ia and Ib, and is presently in a Phase II clinical trial in the Senegal River Delta region in African sites that are endemic for both *S. mansoni* and *S. haemathobium*. It is believed that the development of a safe, effective, and humanitarian vaccine will change the epidemiological profile of this disease, thus freeing countries such as Brazil and much of the African continent from one of the major stigmas of underdevelopment, namely, parasitic diseases, particularly schistosomiasis.

## Figures and Tables

**Table 1:** Sm14 vaccination does not induce changes in pregnancy parameters.

Experimental Groups	Number of Fetuses	Size of Fetuses (cm)	Weight of Fetuses (g)	Number of Corpora Lutea	Number of Placentae (Deployments)	Number of Live Fetuses	Percentage of Live Male Fetuses/Litter
Sm14+GLA-SE	6.60 ± 2.78	6.85 ± 2.40	23.43 ± 11.32	6.33 ± 3.14	7.58 ± 3.02	83	56.25 ± 30.24
PBS Control	7.00 ± 3.24	7.10 ± 0.83	21.58 ± 7.32	7.25 ± 2.89	8.42 ± 2.99	84	55.60 ± 34.91

**Table 2:** Adverse events related to the injection of the vaccine: reactions at the site of injection and systemic reactions during the first 36 h after the administration of the vaccine.

		Proportion (95% Confidence Interval)	
Type of Adverse Event	First Dose (Day 1)	Second Dose (Day 31)	Third Dose (Day 61)
Number of participants	10	10 *	10
Serious adverse events	0.000 (0.000, 0.308)	0.000 (0.000, 0.308)	0.000 (0.000, 0.308)
Reactions at the site of injection Reaction to touch	0.000 (0.000, 0.308)	0.000 (0.000, 0.308)	0.000 (0.000, 0.308)
Erythema	0.000 (0.000, 0.308)	0.000 (0.000, 0.308)	0.000 (0.000, 0.308)
Induration	0.000 (0.000, 0.308)	0.000 (0.000, 0.308)	0.000 (0.000, 0.308)
Edema	0.000 (0.000, 0.308)	0.000 (0.000, 0.308)	0.000 (0.000, 0.308)
Local pain	0.900 (0.555, 0.997)	0.556 (0.212, 0.863)	0.400 (0.122, 0.738)
Involuntary muscle contractions, mild	0.100 (0.003, 0.445)	0.000 (0.000, 0.308)	0.000 (0.000, 0.308)

* One missing value for local pain.

**Table 3:** Secondary adverse events (new symptoms since the last exam) in the ten Sm14-vaccinated women.

Day of Exam	New Symptoms since Previous Exam (Duration)	Relationship to the Sm14 Vaccine
D0 *	#10: Vomit, mild (1 day). #13: Sleepiness, mild (3 days).	Not related to the vaccine.
D7	#7: Fever (2 days). #8: Fever (1 day).	Fever in patients #7 and #8 probably related to vaccine
D30	#12: Gallstones, mild.	Not related to the vaccine.
D37	#10: Local hyperemia, mild (3 days). #13: Light hepatic steatosis; mild headache (1 day each on separate occasions)	Patient #10 probably related to vaccine; #13 possibly related to vaccine.
D60	#3: Cold (6 days). #10: Flu, mild (3 days). #14: Urinary tract infection, mild (3 days).	Not related to the vaccine.
D67	#1: Cervicalgia, mild (unknown duration). #5: Pain in right heel, mild (10 days).	Not related to the vaccine
D90	#7: Vaginal discharge, mild (3 days). #13: Flu, mild (5 days).	Not related to the vaccine
D120	#2: Slight elevation of GGT (1 day). #3: Moderate eosinophilia (1 day). #10: Mild headache (1 day).	Not related to the vaccine

* Previous exam was at baseline. D0 is the day of the first dose. Numbers preceded by # correspond to participants’ code numbers.

**Table 4:** Summary of laboratory tests and physical exams in ten Sm14-vaccinated women.

Type of Test	Tests Outside the Reference Values (RVs) by Participant Number (#) and Day of Occurrence	Toxicity Evaluation
Hematologic tests		
MCV (fl)-RV= (80–100)	#3 at D120 (MCV = 79.7)	No toxicity observed.
Total Leukocytes (TL) (/µL) RV = (3600–11,000) ICTDR G1: 13,000–15,000 /mm^3^	#2 at baseline (TL = 11,170) #10 at baseline (TL = 10,270) #13 at D90 (TL = 11,310) and D120 (TL = 10,940)	No toxicity observed.
Eosinophil (E) (%) RV= (0–5)	#2: at D37 (E = 8), D67 (E = 6), and D120 (E = 6) #3 at baseline (E = 12), D7 (E = 10), D67 (E = 14), D90 (E = 21), and D120 (E = 19) #7 at baseline (E = 8), D7 (E = 12), and D90 (E = 7) #12 at baseline (E = 7), D37 (E = 7), D67 (E = 6), and D90 (E = 6)	ICTDR has no recommendations for toxicity. Elevated values at all follow-ups were probably not related to vaccine.
Neutrophil (%)–RV = (0–5)	No values outside the RV.	No toxicity observed.
Segmented neutrophil (SN) (%) RV = (40–78)	#2 at D90 (SN = 80, NCS) #3 at baseline (SN = 31)	ICTDR has no recommendations for toxicity. Elevated value at D90 was probably not related to vaccine.
Lymphocytes (L) (%) RV = (20–50)	#2 at baseline (L = 16, NCS) and D90 (L = 13, NCS) #7 at D37 (L = 19, NCS) #10 at baseline (L = 17, NCS) and D90 (L = 16, NCS)	ICTDR has no recommendations for toxicity. No toxicity observed.
Atypical lymphocyte	None observed	No toxicity observed.
Monocytes (M) (%) RV = (2–10)	#1 at D120 (M = 13, NCS) #12 at D37 (M = 12, NCS)	ICTDR has no recommendations for toxicity.
Hemoglobin (H) (g/dL) RV = (12.0–16.0)	#2 at baseline (H = 11.6, NCS) #3 at D7 (H = 11.6, NCS), D67 (H = 11.8, NCS), and D90 (H = 11.8, NCS) #5 at baseline (H = 11.6, NCS), D7 (H = 11.4, NCS), D37 (H = 11.7, NCS), D67 (H = 11.6, NCS), D90 (H = 11.4, NCS), and D120 (H = 11.9, NCS) #8 at D7 (H = 11.8, NCS), D37 (H = 11.8, NCS), and D120 (H = 11.7, NCS) #10 at D7 (H = 11.9, NCS) #13 at baseline (H = 11.6, NCS), D7 (H = 11.4, NCS), D37 (H = 11.0, NCS), D67 (H = 11.3, NCS), D90 (H = 11.6, NCS), and D120 (H = 11.3, NCS)	No toxicity observed.
Platelets (/µL) RV = (140,000–400,000)	#14 at D37 (Platelets = 438,000, NCS)	No toxicity observed.
Red blood cells (RBC) (M/µL) RV = (3.9–5.3)	#5 at baseline (RBC = 3.84), D7 (RBC = 3.79), and D90 (RBC = 3.85) #10 at D7 (RBC = 3.87)	No toxicity observed.
Hematocrit (HM) (%) RV= (36–48)	#3 at baseline (HM = 35.4), D7 (HM = 35.9), and D67 (HM = 35.5) #5 at baseline (HM = 34.6), D7 (HM = 34.1), D37 (HM = 34.6), D67 (HM = 35.4), and D90 (HM = 34.3) #8 at D7 (HM = 35.9), D67 (HM = 35.9), and D120 (HM = 34.0) #10 at D7 (35.3) #13 at baseline (HM = 35.2), D7 (HM = 33.5), D37 (HM = 32.4), D67 (HM = 34.0), D90 (HM = 35.8), and D120 (HM = 32.9)	No toxicity observed.
Basophil (%)–RV = (0–2), Myelocyte (%)–RV = 0, Metamyelocyte (%)–RV = 0	None found in any participant during the entire study.	
Activated partial thromboplastin time PTT (sec)-RV = (25.1–36.5)	#10 at D67 (PTT = 23.9) #14 at baseline (PTT = 24.3), D37 (PTT = 24.3), D90 (PTT = 23.8), and D120 (PTT = 24.0)	No toxicity observed.
Prothrombin time (sec) RV = (9.4–12.5)	No values outside the RV.	No toxicity observed.
Prothrombin ratio (PR) (%) RV = (70–100)	#2 at baseline (PR = 101, NCS), D7 (PR = 117, NCS), D37 (PR = 112, NCS), and D67 (PR = 113, NCS) #7 at D7 (PR = 103, NCS), and D90 (103, NCS) #10 at baseline (PR = 104, NCS), and D67 (PR = 101, NCS) #12 at baseline (PR = 104, NCS) #13 at baseline (PR = 112, NCS) #14 at baseline (PR = 115, NCS), and D7 (PR = 114, NCS), D37 (PR = 106, NCS), D90 (PR = 108, NCS), and D120 (PR = 115, NCS).	No toxicity observed.
International Normalized Ratio (INR)–RV = (1.0–1.2)	No values were above the LR RV.	ICTDR has no recommendations for toxicity. No toxicity observed.
Blood Chemistry		
Sodium (mmol/L) RV = (136–145)	No values were above the RV.	No toxicity observed.
Potassium (K) (mmol/L) RV = (3.5–5.1) ICTDR G1: 5.6 to 6.0 mEq/L ICTDR G2: 6.0 to 6.5 mmol/L.	#1 at D37 (K = 5.4, NCS) and D67 (K = 5.7, G1). #10 at D37 (K = 5.2, NCS).	#1 did not show other symptoms or other abnormal laboratory findings and was considered to have no toxicity, according to medical chart.
Liver function		
Total bilirubin (mg/dL) RV ≤ 1.2	No values were above the RV.	No toxicity observed.
Direct bilirubin (DB) (mg/dL) RV ≤ 0.3	#1 at baseline (DB = 0.34)	No toxicity observed.
Indirect bilirubin (mg/dL)–RV ≤ 0.7	No values were above the LR RV.	No toxicity observed
Alkaline Phosphatase (AP) (U/L) RV = (35–104) ICTDR G1 Lower Limit = 130	#12 at D90 (AP = 105) #13 at baseline (AP = 119), D7 (AP = 118), D37 (AP = 106), D67 (AP = 106), D90 (AP = 123), and D120 (AP = 104)	No toxicity observed according to medical charts. High values for #13 are probably not due to vaccine (was high at baseline).
AST (U/L)–RV ≤ 32	#12 at D90 (AST = 37, NCS)	No toxicity observed.
ALT (U/L) RV ≤ 33 ICTDR G1: (40–80)	#12 at D90 (ALT = 66, G1, NCS) #13 at D37 (ALT = 35), D67 (ALT = 35), and D90 (ALT = 33)	#12 above ICTDR threshold for grade 1 toxicity, but not clinically significant according to medical chart. No other toxicity observed.
GGT (U/L) RV ≤ 40 ICTDR G1: (50–100) ICTDR G2: (101–200) ICTDR G3: (201–400) ICTDR G4: > 400	#2 at baseline (GGT = 52, G1), D7 (GGT = 50), D37 (GGT = 58, G1), D67 (GGT = 57, G1), D90 (GGT = 68, G1), and D120 (GGT = 69, G1) #12 at baseline (GGT = 99, G1), D7(GGT = 106, G2), D37(GGT = 113, G2), D67(GGT = 95, G1), D90 (GGT = 240, G3), and D120 (GGT = 107, G2) #13 at baseline (GGT = 122, G2), D7(GGT = 123, G2), D37 (GGT = 112, G2), D67(GGT = 106, G2), D90 (GGT = 130, G2), and D120 (GGT = 102, G2)	Three participants had elevated GGT at baseline and all follow-ups, which were most likely not due to the vaccine.
Kidney function		
Creatinine (C) (mg/dL) RV= (0.5–0.9) ICTDR G1: 0.99–1.35	#13 at D67 (C = 0.95) and D120 (C = 0.94)	No toxicity observed.
Urea (mg/dL) RV < 50 (for age ≤ 65)	No values were above the RV.	No toxicity observed.
Other blood tests		
Calcitonin (pg/mL) RV ≤ 11.5	No values were above the RV	No toxicity observed.
Abdomen physical exam		
Abnormalities	None observed.	No toxicity observed.

# = Participant’s study number. RV = Reference value considered normal by the Richet Laboratory. ICTDR = International Centers for Tropical Disease and Research. ICTDR toxicity grades: G1 = Mild-transient or mild discomfort (<48 h), with no medical intervention or medication required; G2 = Moderate-mild to moderate limitation in activity, with some assistance possibly needed, but no or minimal medical intervention or medication required. NCS = Not clinically significant according to the medical chart and medical interpretation.

## Data Availability

All data that were analyzed during this study are included in this published article.

## Supplementary Materials

The following supporting information can be downloaded at: https://www.mdpi.com/article/10.3390/vaccines10101724/s1. Table S1: Demographic characteristics of the female participants at the baseline (DOCX). Figure S1: Flowchart for the Phase Ib clinical trial, including enrollment and clinical follow-up at the Brazilian National Institute of Infectology Evandro Chagas. (TIF)
