# Supplementary material for: Development of the Sm14/GLA-SE Schistosomiasis Vaccine Candidate: An Open, Non-Placebo-Controlled, Standardized-Dose Immunization Phase Ib Clinical Trial Targeting Healthy Young Women
Source: Vaccines (Basel). 2022 Oct 15;10(10):1724. doi: 10.3390/vaccines10101724 (PMC9607179; doi:10.3390/vaccines10101724)
Supplement: Supplementary file 1 [file vaccines-10-01724-s001.zip › Table S1.pdf]

Supplemental Table S1.
Demographic characteristics of the female participants at the baseline

	Mean (Standard deviation)	Median (Minimum – Maximum)	% (n = 10)
Age (in years)	36.3 (6.8)	36 (26, 48)	
Race*			
Caucasian			20 (2)
Black			10 (1)
Multiple ethnicities			70 (7)
Smoking,% using or used **			40 (4)
Alcohol consumption†			0 (0)
Use of illegal drugs††			10 (1)
Recent vaccination			
No			90 (9)
Yes			10 (1)
Previous disease/condition§, % yes			80 (8)
HIV, % negative			100 (10)

n = number of participants
** One participant had stopped smoking 8 years prior to study
†No participant reported alcohol addiction.
†† Participant reported the use of cocaine for 2 months but has stopped one year before inclusion in the study.
§ List of concomitant diseases/conditions:

- cervix conization due to neoplasia (2010), light cutaneous rash (2013), hypermenorrhea (2011), twisted ankle with light pain and edema (2013), tubal ligation (1995), menstrual cramps (2011 active)
- allergic rhinitis (2013), tubal ligation (2010), monthly menstrual cramps
- tubal ligation (2005), surgical procedure for leg varicose veins (2013)
- sinusitis due to allergy (2013)
- light erysipelas (2013), repeated erysipelas, light (2011), removal of breast fibroma (1998)
- tubal ligation (1995), menstrual cramps (1995)
- Chronic arthrosis of the knee, bilateral (2010)
- Unspecified convulsions (2003, inactive), pharyngitis, light (2013, active)
