# Supplementary figures and images for: Development of the Sm14/GLA-SE Schistosomiasis Vaccine Candidate: An Open, Non-Placebo-Controlled, Standardized-Dose Immunization Phase Ib Clinical Trial Targeting Healthy Young Women
Source: Vaccines (Basel). 2022 Oct 15;10(10):1724. doi: 10.3390/vaccines10101724 (PMC9607179; doi:10.3390/vaccines10101724)

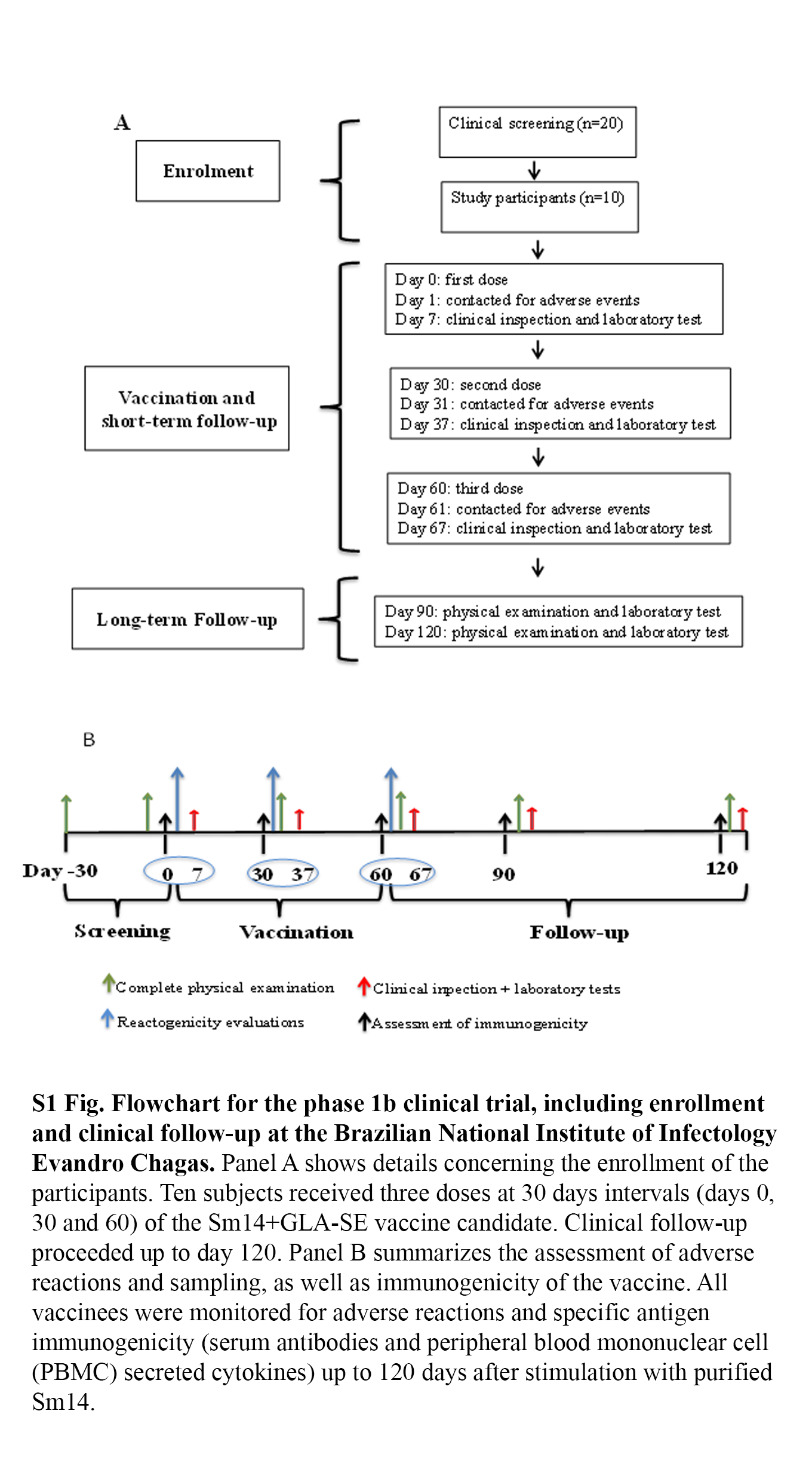

Supplement: Supplementary file 1 [file vaccines-10-01724-s001.zip › Figure S1.tiff]
